# R425 first year student nurses ‘experience of encounters with death of a patient during clinical placement

**DOI:** 10.1186/s12912-024-01922-z

**Published:** 2024-04-16

**Authors:** Lebogang Molefe

**Affiliations:** Sefako Makgatho Health Sciences University, Garankuwa, Pretoria South Africa

**Keywords:** Clinical placement, Death, Encounter, Experience, Patient, R425 student nurses

## Abstract

**Background:**

In the course of caring, nurses often experience the death of patients, and this experience has an effect on the nurse. Every nurse responds to this experience in a different way, and it can be either a negative emotional response, or a positive emotional response. As part of their curriculum, R425 first-year student nurses are placed in clinical facilities to acquire competency in nursing skills, and here they may be exposed to patients dying. R425 is a South African Nursing Council regulation relating to the approval of and the minimum requirements for the education and training of a nurse (General, Psychiatric, and Community) and Midwife, leading to registration. End-of-life care can be rewarding, yet emotionally and psychologically challenging. Little is known about R425 first-year student nurses’ experiences of patients dying while being cared for by nurses on clinical placement. The study, therefore, explored and describes R425 first-year student nurses’ experiences of the death of a patient during clinical placement.

**Method:**

A qualitative exploratory descriptive and contextual research design was adopted, and a purposive, nonprobability sampling approach applied. Data were collected through unstructured individual interviews with 15 R425 first-year student nurses. Data were analysed using content analysis.

**Results:**

Four themes emerged, namely, knowledge, psychological trauma, low self-esteem, and nutritional disorders, and subthemes were identified. Results reveal both negative and positive responses to encountering the death of patients, with more negative responses, and fewer positive responses.

**Conclusion:**

Results show that first-year student nurses struggle to cope with the death of a patient, mainly because they lack knowledge and the skills required to provide end-of-life nursing. It is the requirement for student nurses to be competent in a skill, ‘last office’, which involves laying out of a dead person. Such skill can be deferred in the first year of study, and can only be introduced at a later stage, either in third year or fourth year of study, when students are better equipped with knowledge and skills relating to dealing with death. There is a need to review the curriculum of R425 first-year student nurses, so that outcomes such as death and dying can be introduced in the third or fourth year of study.

**Supplementary Information:**

The online version contains supplementary material available at 10.1186/s12912-024-01922-z.

## Background

To acquire competencies and clinical skills, it is mandatory for student nurses to be placed in clinical facilities as outlined in the Nursing Act No. 33 of 2005 [1]. Furthermore, this is a requirement of the South African Nursing Council (SANC), which oversees the training of all categories of student nurses, and ensures safe nursing practices of all nurses [2]. Under Regulation R425 (the curriculum that was being phased out), the curriculum for first-year student nurses was congested, and comprised many modules. The congestion meant student nurses spent less time with clinical educators, preceptors, and mentors; one of the goals of this contact had been to ensure that students were adequately prepared to deal with death of patients in clinical facilities. Consequently, nursing students find it difficult to cope with deaths of patients because they have not yet been adequately prepared to deal with it, and they lack the ability to adjust psychologically when faced with a patient’s death [3]. When students start their clinical practice, their roles change, from students to caregivers of dying patients, and they become emotionally affected by the situation, and lack the necessary skills to cope with death and dying [4]. Student nurses report being afraid and stressed when dealing with death, because they do not know what to do when faced with the death of another person [5]. The most common emotions of student nurses associated with the death of a patient are reported to be compassion, sadness, and helplessness [6]. Researchers report that high levels of stress and strong emotions are triggered when nurses care for dying patients [7]. These strong emotions could encourage students to seek dysfunctional coping strategies, which include behaviour such as drinking alcohol, or even abandoning their studies [8].

The literature above affirms that the first year of training seems to be an early time to place students in facilities where they will inevitably encounter the death of patients. At that time, students are still fragile, and lack the knowledge and skill necessary to provide end-of-life care. Skills such as effective communication, and ability to cooperate with others, providing emotional support, and compassionate care are key to patient care [9], and are still lacking in first year student nurses. It is for this reason that students become negatively affected when they encounter the death of a patient. The negative experiences are exacerbated by poor communication with patients and their families, lack of knowledge of what constitutes a good death, inadequate mentorship, and poor support. There is, therefore, a need for development of high-quality mentorship, supervision, and promotion of open communication regarding the emotional challenges involved in delivering end-of-life care [7]. Against this background, it is proposed that the ideal time to place first-year student nurses in facilities where they are likely to encounter patients dying would be their third and fourth years of study; by that time, students have more experience, and have developed strategies to cope with death, which first-year student nurses have not been able to do yet [10].

Based on the background, it is evident that first-year student nurses feel unprepared to care for dying patients, and to handle patient death. Understanding the experiences of first-year student nurses in dealing with death and dying of patients could inform how palliative care education can be improved, and how student nurses can be supported better in clinical settings. Little is known about R425 first-year student nurses’ preparedness for taking an active role in caring for patients who are dying, or their ability to manage challenges associated with caring for dying patients and their death. The study, therefore, sought to explore and describe R425 first-year student nurses’ experiences of encountering death of patients during clinical placement. An in-depth understanding of the experiences of R425 first-year student nurses in this situation will provide insight into their emotional responses to such experiences, and student nurses’ need for support, including psychological support. Thereby, the incidence of early termination of training can be prevented, and the gap between the theoretical knowledge offered by a nursing programme and its application in professional practice can be closed. There seems to be a need to review the curriculum for first-year nursing students.

## Methods

The researcher adopted a phenomenological approach to explore the lived experiences of first-year student nurses who have to deal with the death and dying of patients. A phenomenological approach is a type of qualitative research that studies individuals’ lived experiences in the world [11]. A phenomenological approach seeks to obtain indepth, contextualised, open-ended responses from research participants about their views, opinions, feelings, knowledge, and experiences [12]. The approach was found to be appropriate, because it helped the researcher to understand the experiences of R425 student nurses in dealing with the death of patients [13]. A qualitative exploratory descriptive and contextual research design was undertaken. This type of design is used when a researcher wishes to connect to the research problem to achievable empirical research [14], by exploring a topic with limited coverage by the literature. This approach means that participants can contribute enormously to the development of new knowledge [15].

### Study setting

The study was conducted in Gauteng province, South Africa. Gauteng is the smallest province in size, with the largest population and highest number of student nurses in training with the R425 nursing curriculum. The R425 nursing curriculum for first-year student nurses consists of the following modules/subjects: Fundamental nursing science, Ethos and professional practice, General nursing science, Biological and natural science, and Pharmacology and social science. The intention is that the various fields of study are integrated in student nurses’ clinical application. Fundamental nursing science, Ethos and professional practice, General nursing science, Pharmacology and social science are modules that provide knowledge and skills pertaining to patient care, including dealing with death and dying of patients. The academic period of 44 weeks in a calendar year is used to ensure that students are competent in the topics of all these modules [2].

An accredited public nursing college in Gauteng was selected for this study, because the problem under discussion was first identified at this establishment. The researcher, who is a woman who has five years’ experience of teaching and 16 years’ experience of providing care to patients, was a lecturer at a nursing college, where she facilitated the theoretical component of a module. The relationship between the researcher and participants was, therefore, a lecturer–student relationship. The college has clinical lecturers who facilitate clinical components, and who accompany students during clinical placements to mentor, guide, and support students. The researcher, as the lecturer, therefore, never had encounters with students during their clinical placement. There were in total 400 first-year students, of whom 300 were females and 100 males. The academic year lasts 12 months, from January to mid-December. In these 12 months, students spend six months in clinical facilities to acquire clinical skills competencies, and the other six months at the college, to gain theoretical competencies. In January, when the academic year starts, students are divided into groups, which alternate between clinical work and academic study (the latter broadly termed ‘theory’), so that the large number of trainees can be accommodated in the available space, and an even distribution can be arranged between the two aspects of training, thus, securing equal opportunities to receive all aspects of training across the board, despite the material constraints of available facilities. To acquire competency in clinical skills and hours expected by the SANC, students are placed at hospitals, homes for the aged, hospices, and rehabilitation centres. However, the majority of expected hours are spent in hospitals, and it is during clinical exposure in hospitals that students experience death and dying of patients. In reflection journal reports, all first-year students affirmed having been exposed to death and dying of patients. In these reports, students communicated the burnout and stress they experienced from encountering death and dying of patients in clinical facilities.

### Participants

All students were approached in their groups to request that they participate in the study, and the study, its purpose, risks and benefits, and that participation would be voluntary, were explained to them. Participants were selected if they would serve as information-rich cases that contributed to the central focus of the study, hence, a purposive, nonprobability sampling approach was used [16]. The population comprised first-year student nurses who were registered for the R425 nursing curriculum. Because participation was voluntary, only first-year student nurses who agreed to participate were selected. Initially, from the group of 400 students, only nine students agreed to participate– seven women and two men.

These nine students were interviewed. In this round of interviews, saturation was reached with the fifth participant. In other words, no new information was forthcoming in the interviews with the sixth to ninths participants. The study formed part of a Master’s degree, and both the researcher and her supervisors were satisfied with the data collected. They agreed that the researcher could proceed to report the data in a dissertation, which was examined, approved and accepted. Upon enquiring about submitting a manuscript for publication in mid-October 2012, it was decided that the data was not sufficient for an article. The researcher, therefore, collected data collection from another six participants who agreed to participate. The researcher used the same ethical clearance certificate that had been issued in March 2012, as it was still valid (less than a year had passed), and the title of the research was unchanged.

Therefore, a total of 15 first-year student nurses were interviewed by the researcher. All participants were at least 18 years old, and they signed voluntary consent forms. Inclusion criteria were all first-year student nurses at the chosen nursing college who agreed to participate, and had experienced the death of a patient during clinical placement in hospital wards. Exclusion criteria were student nurses in their second, third and fourth years of study. Participants’ rights to privacy and confidentiality were respected, hence, each participant was assigned a code as a pseudonym. Table 1 provides the demographic information of participants.

**Table 1:** Demographic information of first-year student nurses who participated in the study

Participant No.	Age (Years)	Gender
P-A	24	M
P-B	19	M
P-C	30	F
P-D	20	F
P-E	21	F
P-F	32	M
P-G	23	M
P-H	24	F
P-I	20	F
P-J	21	F
P-K	19	M
P-L	29	F
P-M	22	M
P-N	26	F
P-O	31	F

### Data collection

Interviews are the most versatile method to collect data in qualitative research. Interviews can be structured, semistructured, or unstructured, and it consists of organised, predetermined open-ended questions that are flexible, and may be modified according to the situation and response. This study used unstructured interviews to collect data. It allowed participants to openly reply to questions related to the topic. An interview is a subjective, detailed and direct verbal method used to elicit detailed narratives from participants [12]. The unstructured questionnaire was developed by the researchers and had not been used before. All participants agreed to be interviewed in the language used for teaching and learning, which is English. The researcher reassured participants that their rights, well-being and safety would take precedence over research objectives. The researcher requested permission from participants to audio record the interviews, and all participants agreed. Participants were interviewed at different dates and times, as decided by the participants. The first nine participants were interviewed between 15 January 2012 and 3 September 2012. The second round of interviews, with six participants, took place between mid-October 2012 and midDecember 2012, using the very same ethical clearance, as it was still valid. The same procedure was followed. No new information was obtained from the six participants who were interviewed in the second round.

Interviews were in vacant classrooms; a ‘Do not disturb’ sign was placed outside the venues. The researcher did not rush the interviews, and arrived 30 min before the scheduled time to make the necessary preparations, and set aside an hour after the interviews to record fieldnotes. In total three hours was allocated overall per participant to complete the interview. The researcher ensured that the interviews were conducted in a stress-free and unhurried fashion. Participants had been contacted seven days before the interviews to confirm the location and the interviewee’s commitment to attend the interview at the venue at the time agreed upon, as well as to give interviewees an opportunity to ask questions about concerns they might have. On the day before the scheduled interview, the researcher checked the audio recorder to ensure that it was fully functional. During the interview, the researcher did not face the interviewee directly, as this could have been seen as confrontational. The researcher took pains to build rapport with participants by explaining the purpose of the study, how long the interview would take, what the data would be used for, that confidentiality would be guaranteed, and that participants were free to withdraw from the interview at any time without facing penalties.

The following questions was asked unambiguously and in a neutral tone: *How did you experience dealing with death of a patient during clinical placement?*[AA]. Participants were encouraged to report their experiences and encounters, the researcher avoided interrupting. The researcher made use of the following probing questions to achieve better understanding and greater clarity: *Have you only had to deal with one patient’s death/dying in your clinical experience? Were there differences between the first and subsequent experiences? Explain* [AA]. In addition, the researcher interpolated with the following questions: *What support did you get in the ward? How did you feel? What do you think could have been done to relieve the situation?* [AA]. The researcher made use of communication skills such as nodding, reflection (i.e., pausing thoughtfully) and maintaining eye contact, and recorded fieldnotes relating to non-verbal communication, such as physical appearance, manner of speaking, style of interaction and emotional reactions. Interviews lasted between 30 and 50 min; no interview exceeded one hour.

### Data analysis

Content analysis was used to analyse data. During the first round of data collection from the nine participants, a co-coder was not used, because the researcher was working with her supervisors to complete a dissertation. Themes and subthemes that were identified were confirmed by a supervisor, and were subsequently recorded and accepted as the final themes and subthemes of the dissertation. Content data analysis assisted the researcher to extract essential meanings of the topic from the recorded data, separate the meanings into constituent concepts, then arrange them into themes and subthemes [17]. The following steps of content analysis suggested by Creswell and Creswell [18] were used.

#### Transcribe interviews

The researcher listened to the audio recording and transcribed and documented the data. A separate file was created for each participant. Every interview was documented separately in numerical order with a front sheet reflecting the date, location and time of the particular interview, as well as the participant’s code number. Flavours and behaviours expressed in participants’ words, facial expressions, gestures and reactions generally were captured. To avoid the possibility of disturbing the flow of the interview and, therefore, a participant’s authenticity, notes were recorded immediately after the interview by the researcher, in the participant’s presence, and are duly reflected in the researcher’s analytical memos.

#### Organise, order and store data

Details of time, location and attendant comments were recorded on all transcripts and fieldnotes. Data were recorded, cross-checked and labelled. All materials and files were stored in a locked closet to ensure safety and security. At first, horizontal pass was used for the data analysis, because it is more holistic. Data was read, and themes, emotions and surprises were considered. Reflective and in-depth reading of the data was done to find supportive evidence for themes. Data was reread to identify elements that might have been overlooked. Then, the researchers searched for possible alternative meanings and attempted to link discrepancies.

#### Listen to and read collected data

An analytical style was used to reflect on each transcript, search for and record significant statements, then deleting repetitive and overlapping statements so that only invariant constituents of the phenomenon remained, which were organised into themes. Verbatim quotes from the data were included to capture the texture of the experience as recounted by the participants. Finally, a description of the meanings of the experience was developed.

#### Code and categorise

Coding was used to explore the data and single out words used by participants, to prevent the researcher’s own frame of reference/perceptions and ideas being imposed on the data. The researchers resorted to open coding, which entailed labelling specific pieces of data.

#### Build themes

Researcher started with a mass of codes that were reduced until each one represented a specific concept. Coding was done paragraph by paragraph. The researcher took care to avoid imposing preconceptions and, instead, deferring rigorously to ideas emerging from analysis of the data provided by participants. Each note in a transcript was read and reread, observations and experiences were recalled, and the audio recordings were replayed to ensure familiarity with the information disclosed by participants. Tone and emphasis were gauged from listening to audio-recorded interview data. The researcher made use of data reduction to reduce the volume and thereby reducing list of themes.

Themes and subthemes were identified and presented in a cohesive manner, and interpreted to produce findings. After a second round of data collection, which was not part of the original dissertation, the researcher involved a co-coder in the data analysis, because, at that time, the researcher was no longer registered for a Master’s degree, and a co-coder could ensure that bias was avoided. Furthermore, ideally, coding should be performed by at least two researchers, especially at the beginning of the coding process. Both the researcher and the co-coder applied content analysis, following the same process as explained above; however, the co-coder analysed the data independently. The researcher and co-coder discussed the themes they had identified. There were similarities in the themes and subthemes, and one subtheme about which the researcher and co-coder disagreed. During an online meeting on 10 April 2013, they engaged in deliberations and reached consensus on the final themes and subthemes on the same day.

#### Trustworthiness

Five testing criteria were observed: credibility, dependability, confirmability, transferability, and authenticity [11]. Credibility was attained through accurate identification and description of participants, prolonged engagement with participants, persistent observation throughout the data collection process, recording fieldnotes, member checking, triangulation, and verbatim transcription of interviews. Dependability was attained through description and application of the research methodology, providing an audit trial, and involving a co-coder during analysis of data. Confirmability was attained by ensuring that researcher bias, motivation, or interest did not shape the findings– this step involved doing bracketing and triangulation, and involving a co-coder. Transferability was attained through thick descriptions of the research methodology, and triangulation. Authenticity was attained by ensuring that participants who provided rich information pertaining to the topic were selected. During reporting of the findings, the researcher ensured that the report represents only the feelings of participants, not the views nor feelings of a researcher.

## Results

Themes and subthemes were identified during data analysis and will be discussed in this section. Table 2 is a summary of themes and subthemes.

**Table 2:** Themes and subthemes

Themes	Subthemes
Knowledge	• Lack of knowledge • Denial • Empowerment
Psychological trauma	• Terrified • Stressed • Insomnia
Low self-esteem	• Feeling worthless • Feeling of not belonging • Loss of confidence
Nutritional disorder	• Lack of appetite

### Theme 1: knowledge

Knowledge is important if a specific skill is to be performed competently. Knowledge is a fluid mix of framed experiences, values, contextual information, expert insight, and grounded intuition that accumulates over time in people’s minds and that provides an environment and framework for evaluating and assimilating new experiences and information, thereby, expanding the existing store [19]. A common assumption is that we build our storehouse of world knowledge through direct experience. Although direct experience is involved in building knowledge, self-derivation through an integration paradigm provides a valid model for how we build semantic knowledge, including observations that performance on the task correlates with and predicts individuals’ world knowledge and academic success [5]. In this instance, knowledge includes the first-year student nurses’ experiences of their encounters with the death of a patient during the students’ clinical placement. Therefore, the researcher ensured that only student nurses who had encountered the death of a patient participated in the study. Subthemes for knowledge are lack of knowledge, denial, and empowerment.

#### Lack of knowledge

Lack of knowledge refers to the result of a failure to assimilate facts, information and skills that are deemed necessary for a particular purpose. Lack of knowledge contributes to inflating a person’s assessments of how much they know, and contributes to a person’s unintentional ignorance of risks [20]. In this instance, participants– first-year student nurses– lacked knowledge for dealing with the death of patients in clinical facilities. The following quotes attest that they did, indeed, lack knowledge:

*I had no knowledge of how to manage a dead patient. When I ask a sister, she just shut me out without advising me on what to do. Sisters shut us out when we ask things (P-N).*

Lack of knowledge makes a person unsure whether the act they are performing is the right one. Even if the person has a cognitive idea, they are unsure whether it will yield positive results when it is applied practically. Without knowledge, a person find themselves in darkness, not knowing what to do, and not sure of anything:

*I and my colleague were arguing if the patient is dead or not because we were not sure. Even though we were taught signs of a dead person at college, we were not sure. We did not know what to do (P-C).*

*We did not know that the patient has passed away, we just continued to bath him. We also did not know how to tell the relatives that the patient has died (PF).*

#### Denial

Denial is a defence mechanism by which an individual refuses to recognise or acknowledge objective facts or experiences, A person who is in denial is trying to protect themself from a truth that is painful to accept [21]. In the case of this study, denial was often attributable to inadequate knowledge and information about death and dying. On the part of participants, denial was evident from their feedback.

*I kept on arguing with my colleague that the patient has pulse, I guess I was in a denial (P-B).*

*To me, the patient was just having fits, only to find that he was actually dead, and I did not want to accept that he is dead (P-F).*

#### Empowerment

Empowerment is a multidimensional social process that helps people gain control over their lives, thereby fostering power that extends their capacity to deploy measures that enable them to gain advantages in their interaction with others [13]. Empowerment enabled first-year student nurses who experienced the death of patients during clinical placement to gain advantages from their interaction with a dying patient, and ultimately handling a dead patient. The encounter increased their autonomy and coping skills. Participants’ quotes provide evidence of this subtheme.

*Although I had to deal with death without adequate knowledge, I have learned a lot. The patient died from bacterial meningitis, and now I know what bacterial meningitis is, what are the signs and management of it, because I had to do more research regarding the condition after death. I am confident that I will get a distinction in examination if the question about bacterial meningitis can come out (P-L).*

*I am no more ignorant. I am now empowered. Now I know what to do. I can now treat patients with respect and dignity (P-M).*

### Theme 2: psychological trauma

Psychological trauma relates to anything that overwhelms an individual’s ability to process and integrate psychologically something that has happened to them– a traumatic event. Such an event can lead to a sense of powerlessness and can disrupt beliefs and expectations when an individual loses control over the situation and becomes a victim of the circumstances [22]. Psychological trauma is an emotional response that is caused by severe, distressing events that are outside the normal range of human experiences. The event must be understood by the affected person, and the experience can be direct (for example, rape) or indirect (for example, watching a horror movie, or seeing a ghost or a dead person). The event can be extremely distressing, and can produce an involuntary and possibly overwhelming stress response [23]. Subthemes for psychological trauma are terrified, stressed and insomnia.

#### Terrified

A terrified person is fearful or scared. The word terrified implies overwhelming, often paralysing fear. Fear and anxiety is something that can be handled when it is experienced now and then, but when it becomes severe and long-lasting, it can cause a mental health problem [24]. Participants’ encounters of the death of patients in the wards resulted in severe and long-lasting fear; even weeks after an encounter, they were still terrified. Participants’ quotes affirm that encountering the death of a patient was an indirect event that resulted in psychological trauma, because it the effect was too deep and lasted for too long:

*As I close the eyes of a patient who according to me was dead, one relative told me that I was not supposed to do that. I became terrified. Even now, I am now scared to be alone as I keep on seeing the face of that patient (P-B).*

*Every time I lay a female corpse, I become more scared, thinking: what if I can lose my mother? (P-G).*

*For an exceedingly long time after, I had flashbacks of the patient. It was very traumatic (P-I).*

*I begged the sister not to let me lay the corpse again. I was so scared and terrified. Even now I am terrified. (P-K).*

#### Stressed

Stress refers to a disruption of meanings, understanding and smooth functioning, Such disruption can cause harm to a person’s holistic being. Stress is a set of physiological, psychological and behavioural reactions that serve an adaptive function. With stress, a person can experience loss of already-acquired skills, and no acquisition of new skills can occur [25]. Stress is not normally considered a mental health problem, though it is connected to mental health; stress can cause mental health problems. For example, if a person experiences a great deal of stress, they can develop mental health problems such as anxiety, depression, or post-traumatic stress disorder [25]. Stress reactions are exemplified in the following observations made by participants on the stress they experienced after a patient died:

*I was stressed, I could not even be allowed to cry because I was told that nurses are not supposed to cry (P-A).*

*It was hurting and stressing to see that the patient is no more alive. I felt like quitting the course. It was too much to manage (P-F).*

*Days after, I started to have panic attacks. I had to be referred to a psychologist, who prescribed medication to calm me down. I felt like I am losing my mind (PJ).*

#### Insomnia

Insomnia is a sleep disorder that makes it difficult for a person to fall asleep or stay asleep. A person may also wake up too early and struggle to get back to sleep. Insomnia can result in feelings of frustration, anxiety, and distress, which create negative associations with sleep. Insomnia is a main feature of depression, and can be triggered by stress. Research has found that insomnia can trigger mental health symptoms, hence, it is difficult to separate insomnia from mental health disorders [26]. Participants of this study experienced fear, anxiety and stress when they encountered the death of a patient, as affirmed by their responses in the interviews:

*I think that the patient’s ghost is haunting me because every time I tried to sleep, I see his face (PB).*

*It was extremely hard. I could not sleep. I moved out of nurses’ home so that I can always sleep with my mother. Even when I was in my mother’s bed, I still could not sleep (P-L).*

*I could not sleep at night. I kept dreaming about this patient. I kept hearing his voice saying, ‘why did you kill me’? I killed the patient because I performed some exercises, only to find that I was not supposed to do that because the patient had bacterial meningitis (P-O).*

### Theme 3: low self-esteem

Self-esteem refers to a person’s overall sense of their value or worth; it refers to confidence in one’s own worth, abilities, or morals, and emotional feeling of self-appreciation. Someone with low self-esteem lacks confidence about who they are and what they can do. People with low self-esteem often feel incompetent, unloved, or inadequate. Signs of low self-esteem include saying negative things about oneself, loss of confidence, not believing in oneself, feeling useless, ignoring one’s own achievements, blaming oneself, and thinking that other people are better than oneself [27]. Participants had low self-esteem due to encounters with the death of patients, and they verbalised some of the signs of low self-esteem, which are organised in the subthemes of feeling worthless, feelings of not belonging, and loss of confidence.

#### Feeling worthless

A person who feels worthless believes that they are useless, and see themself as hopeless and insignificant. Feeling worthless, furthermore, plays a role in the alternative revised learned helplessness mode [28], as exemplified by participants in this study, who were of the opinion that they had failed to meet the main goal of the nursing profession, which is to preserve life. They expressed this perception in their responses:

*I felt so useless and guilty for not knowing how a dead person looks like (P-D).*

*I performed passive exercises on the corpse, thinking I am helping the patient, only to find that I am providing care to a person who has died, because I could not even identify that the patient is no more. I felt so useless and insignificant (P-F).*

#### Feeling of not belonging

Belonging is a subjective feeling of deep connection with social groups, physical places, and individual and collective experiences. It is a fundamental human need that predicts numerous mental, physical, social, economic, and behavioural outcomes. The feeling of not belonging, also called estrangement, refers to a climate of separation and exclusion, which leaves a person with trauma and longing [29]. Participants engaged in this study voiced their subjective sense of not belonging to a nursing profession through utterances such as the following:

*I feel that I do not belong to this profession. If I do belong, then why is it that two patients − two patients– died in my care? (P-N).*

*I did not even resuscitate the patient because I did not know how. Had I known the procedure, I would have saved the patient’s life. I feel that I do not belong to the profession, I cannot save life. I want to quit because I do not belong here. (P-K).*

#### Loss of confidence

Confidence refers to the certainty that one possesses the ability to achieve or accomplish a feat of some sort. Loss of confidence, therefore, means the opposite, and it is synonymous with hopelessness, pessimism, dejection, depression, despair, disappointment, discomfiture, dismay, downheartedness, melancholy, sadness, cold feet, low spirit, and ‘the blues’. People who have lost confidence have an unbalanced view of themselves, and doubt their abilities and what they have to offer [30]. Participants in the study expressed their loss of confidence in utterances such as the following:

*I do not feel confident at all. Every time there is a patient who is dying, I make sure that I stay away. I do not feel like a nurse at all. (P-E).*

*Each time I see the screen closed, I will suspect that it is a corpse and I run away because I do not feel confident to handle a dead patient (P-O).*

### Theme 4: nutritional disorder

Nutritional disorder is a physiological condition that occurs when a person’s food intake does not contain the right amount of nutrients for healthy functioning, or when a person cannot correctly absorb nutrients from food [23]. Stress and anxiety can trigger emotional and psychological changes in the body, and these changes often affect the stomach and the digestive tract, and cause a person to lose their appetite. Once the stress or anxiety disappears, hunger returns, and the person can start to eat normally [31]. Nutritional disorder is discussed here as a psychosomatic disorder because its physiological manifestation is largely attributable to emotional factors. Participants of this study experienced stress and anxiety, as discussed, and nutritional disorder, which manifested as a loss of appetite.

#### Lack of appetite

Lack of appetite is a condition characterised by the absence of hunger. It occurs because of a variety of reasons, and can cause nutritional deficiency. It can have a negatively effect on a person’s health, overall well-being, and quality of life [32]. When someone starts to feel stressed or anxious, the body begins to release a stress hormone, corticotropin-releasing factor. This hormone activates the sympathetic nervous system, thus, suppressing appetite [32]. As participants of the study indicated that they experienced stress and anxiety, it is safe to say that the cause of their lack of appetite was stress and anxiety. The following testimony of a participant attests that the condition is a reaction to the stressful experience of the death of a patient:

*I could not eat after the incident. I was even admitted, and the doctor diagnosed me as having post-traumatic nutritional disorder (P-C).*

*Whenever I tried to eat, I will vomit. The picture of that dead patient would always come to my mind. I even lost lots of weight. The doctor was even considering admitting me in the hospital for intravenous therapy (P-L).*

## Discussion

The first theme, knowledge, emphasises that, for a person to perform an activity effectively and efficiently, they must be knowledgeable about what to do and how to do it. Without knowledge, the performance of an activity will yield negative results. It is important that people who are knowledgeable transfer knowledge to those who lack knowledge [29]. Education and training must involve senior healthcare professionals engaging in planning and coordinating teaching activities, developing clinical training on the job, and supervision, until a student is competent to perform a job on their own. Senior professionals, such as lecturers, facilitators and clinical professionals in the wards, have to provide students with knowledge of dealing with the death of patients, because knowledge is key in safe provision of care [33]. However, it is evident that student nurses in this study did not have knowledge and did not receive guidance. The conclusion we can draw is that students were not adequately prepared to deal with the death of patients. Student nurses are often placed in the wards with limited training and inadequate knowledge of how to deal with death, and this compromises their well-being and the quality of care [13], which poses a serious challenge for students. In addition to the negative subthemes related to knowledge, the subtheme of empowerment supports the notion that experiencing something unknown can teach a person to become a better being. It is through experience that one develops knowledge, skills and understanding of how to react in a certain situation, and how to deal with problems [32].

The second theme, psychological trauma, explains the overwhelming impact of dealing with death of a patient, hence, the emergence of the subthemes of terrified, stressed and insomnia. Dealing with the death of a patient can cause feelings of compassion, sadness, and helplessness [26]. Because of their unpreparedness, the consequences of a patient dying were severe for student nurses. The reason why student nurses were more affected than senior professionals, is because they experienced death of patients more often than senior staff, at an early stage of their training when they had just joined the profession, and were still unsure of how to handle patients [8]. It is for this reason that students have to undergo extensive training before they are exposed to patients dying.

The third theme, low self-esteem, refers to a loss of self-value or self-worth, hence, the subthemes of feeling worthless, feeling of not belonging, and loss of confidence. Student nurses, due to their inability to adequately manage the death of a patient, had low self-esteem. People with low selfesteem have extreme self-criticism, think badly of themselves, are extremely critical of themselves, downplay their positive qualities, and judge themselves as inferior [34]. Student nurses felt useless when a patient died while in their care and, furthermore, felt a sense of not belonging to the nursing profession. Students blamed themselves for deaths because, in their view, if they had had adequate knowledge and expertise to handle a dying patient, they might have saved the patient’s life through provision of better care.

The fourth theme, nutritional disorder, emphasises the negative effect of dealing with the death of a patient on student nurses. They could not eat, lost weight and experienced nutritional deficiencies. There is a correlation between appetite and exposure to death. People who encounter the death of someone have lower levels of appetite, are at risk of significant weight loss, and might even need medical treatment to cure the disorder [35]. It is, therefore, understandable that student nurses of this study presented with nutritional disorder.

Because of the qualitative nature of the study, the findings cannot be generalised to other colleges. Therefore, a limitation of the study is that that it was confined to a one nursing college in one province of the country, which means that the experiences of R425 first-year student nurses in nursing colleges in other provinces of the country are unknown. However, the findings of the study could be applicable to other settings in South Africa, as the themes discussed are universal issues, and could be applied to improve the ability of R425 first-year student nurses to deal with the death of patients. Furthermore, the research was conducted in a nursing college in the public sector. Private nursing colleges and nursing schools were excluded, and experiences of first-year student nurses in those institutions are still unknown.

The study makes the following recommendations.

### Nursing practice

Clinical professionals should endeavour to adopt an enthusiastic attitude for mentoring student nurses, especially vulnerable first-year student nurses. Such an attitude will assist student nurses to be competent in their delivery of quality service to patients, and it will boost their self-esteem and morale, thus, enabling them to enjoy the nursing profession. In addition to mentoring, there is, furthermore, a need for student nurses to undergo regular in-service training on dealing with the death of patients. In addition, it is recommended that the Department of Health employs more psychologists and counsellors who can provide counseling, de-briefing, and emotional support to counter the traumatising effects experienced by student nurses after dealing with death of patients.

### Nursing education

Clinical outcomes envisaged for first-year student nurses need to be revised with a view to ensuring that procedures, such as dealing with death, are deferred until students have gained more knowledge, skills, and experience, so that they can deal with patients’ deaths. Furthermore, clinical facilities need to have access to full-time clinical preceptors and mentors who can guide and support students throughout their studies.

### Nursing research

It is recommended that studies on this topic are undertaken at other nursing colleges of the province, private nursing colleges and nursing schools. These studies will assist in determining if experiences are similar, whether there are different strategies to empower students to deal with patient death, and to broaden the research under review.

## Conclusion

The study found that first-year student nurses struggle to cope with the death of patients. The deduction is that skills, such as last offices, should be deferred from the first year of training, and introduced at a later stage, preferably in the third or fourth year of study. At that stage, student nurses should be better equipped with knowledge and skills to handle the death of patients. The conclusion that can be drawn is that there is a need to review the curriculum for first-year student nurses. Results of the study can be utilised by future studies that aim to review the first-year student nurse curriculum, and studies aiming to develop guidelines of support for first-year student nurses in clinical facilities.

### Electronic supplementary material

Below is the link to the electronic supplementary material.

Supplementary Material 1

Supplementary Material 2

## Data Availability

The data supporting the findings of this study are available on request from the corresponding author, LM, upon reasonable request.

## Declarations

### Declarations

The author declares that a valid ethical clearance certificate was obtained prior the study. All sources used are duly acknowledged. Methods were applied in accordance with guidelines.
