# Supplementary material for: R425 first year student nurses ‘experience of encounters with death of a patient during clinical placement
Source: BMC Nurs. 2024 Apr 16;23:246. doi: 10.1186/s12912-024-01922-z (PMC11020469; doi:10.1186/s12912-024-01922-z)
Supplement: Supplementary file 1 — Supplementary Material 1 [file 12912_2024_1922_MOESM1_ESM.docx]

**ANNEXURE (AA): INTERVIEW GUIDE**

Grand tour Question:  *How did you experience dealing with death of a patient during clinical placement?*

Probing Keywords:

-*“What do you mean”,*

*-“Please clarify”*

*- “Please explain more”*

*-“I do not understand”.*
